# Cell Penetrating Thyclotides Facilitate Efficient Delivery of Bioactive Peptides into Cells

**DOI:** 10.64898/2026.07.01.735572

**Published:** 2026-07-02

**Authors:** Gamze Ayaz, Hongchao Zheng, Harsha Amarasekara, Victor Clausse, Andy Tran, Ferenc Livák, Michael Kruhlak, Daniel H. Appella

**Affiliations:** 1 Synthetic Bioactive Molecules Section, Laboratory of Bioorganic Chemistry (LBC), National Institute of Diabetes and Digestive and Kidney Diseases (NIDDK), National Institutes of Health, 8 Center Drive, Room 404, Bethesda, Maryland 20892, United States.; 2 Microscopy Core Facility, Center for Cancer Research, National Cancer Institute (NCI), National Institutes of Health, Bethesda, Maryland 20892, United States.; 3 Laboratory of Genome Integrity Flow Cytometry Core, Center for Cancer Research, National Cancer Institute (NCI), National Institutes of Health, Bethesda, Maryland 20892, United States.

## Abstract

Cell penetrating thyclotides (CPTs) are synthetic molecules that promote highly efficient cellular uptake and endosomal escape of bioactive peptides. While peptides are valuable as medicinal agents, their translation to therapies is often limited by their inability to cross cell membranes. CPTs have a unique combination of chiral tetrahydrofurans and polar sidechains within a molecular scaffold that can be optimized to efficiently deliver peptide cargo into cells. The cellular uptake and endosomal escape of two peptides with anticancer biological activities but low bioavailabilities were remarkably improved after conjugation to a CPT. Using CPTs to overcome barriers to cellular uptake represents a new direction for the intracellular delivery of bioactive molecules, and will accelerate drug development for new medical therapies.

Intracellular delivery of bioactive molecules is a difficult barrier for many drugs with therapeutic potential.^1,2^ While drugs with molecular weights below 500 Daltons may pass through cell membranes by passive diffusion, larger and more complex molecules often rely on delivery vehicles to access the interior of a cell.^3^ A wide range of delivery vehicles have been investigated, such as polymers, nanoparticles, liposomes, viruses, and cell-penetrating peptides.^4^ These approaches involve combining a biologically active molecule (the cargo) with a delivery vehicle that in many cases results in the vehicle plus cargo being actively transported into an endosome.^5^ Even though the endosome is inside the cell, the molecular cargo must subsequently escape from the endosome and enter the cytosol to exert biological activity.^6^ A common shortcoming of delivery vehicles is that most of the molecular cargo remains trapped inside endosomes where it remains inactive and may ultimately be degraded.^7^ This shortcoming is particularly problematic when the molecular cargo is an α-amino acid polypeptide (a peptide). Techniques to design and identify peptides that potently bind to and inhibit a biological target *in vitro* are highly successful and continue to rapidly improve with the use of artificial intelligence.^8^ Unfortunately, most peptides are unable to enter cells.^9^ To improve the bioavailability of peptides, common strategies include chemical modification with unique amino acids,^10,11^ cyclization via stapled peptides^12^ or related strategies,^13-19^ or the attachment of cell penetrating peptides.^20,21^ These strategies frequently rely on the presence of multiple positive charges from primary amines or guanidines, and cell penetrating peptides commonly have multiple lysines and arginines in their sequences. While cell penetrating peptides can promote cell uptake of peptide cargo, these types of peptides are plagued by problems of stability, non-specific toxicity to healthy cells, and lack of endosomal escape that limit their use for drug development.^22,23^ A completely synthetic molecular scaffold that replaces cell penetrating peptides could overcome these problems and significantly enable the *in vivo* translation of bioactive peptides. However, the synthetic versions of cell penetrating peptides continue to rely on multiple guanidine groups for cell uptake which lead to non-specific toxicity and endosomal entrapment.^24-27^ Identifying new chemical groups that lead to cellular uptake remain very challenging, and there is currently no synthetic platform that can be used as replacement for cell penetrating peptides.

## CPTs enhance the uptake of target peptides for cancer cells

**Figure 1.**
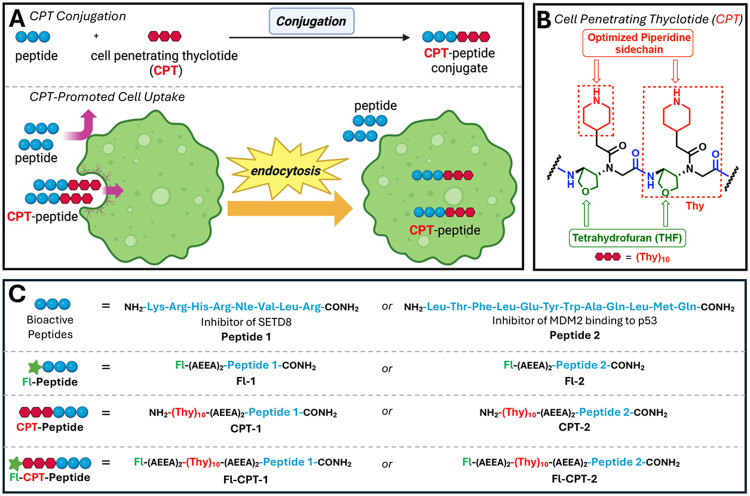
Design and schematic structure of cell penetrating thyclotide (CPT) conjugates. **(A)** Schematic illustration of the proposed cellular uptake mechanism of CPT-peptide conjugates. Following conjugation to cargo peptides, the cell-penetrating thyclotide (CPT) facilitates intracellular delivery into cancer cells primarily through endocytosis-mediated uptake pathways. **(B)** Chemical structure of the CPT monomer used for peptide conjugation. The structural features responsible for cellular internalization and enhanced solubility are highlighted. **(C)** Schematic representation of the peptide conjugates and their corresponding fluorescein-labelled derivatives used in this study. Peptide sequences, CPT-conjugated constructs, and fluorescein (FL) labeled molecules employed for cellular uptake, localization and functional analyses are shown.

**Figure 2.**
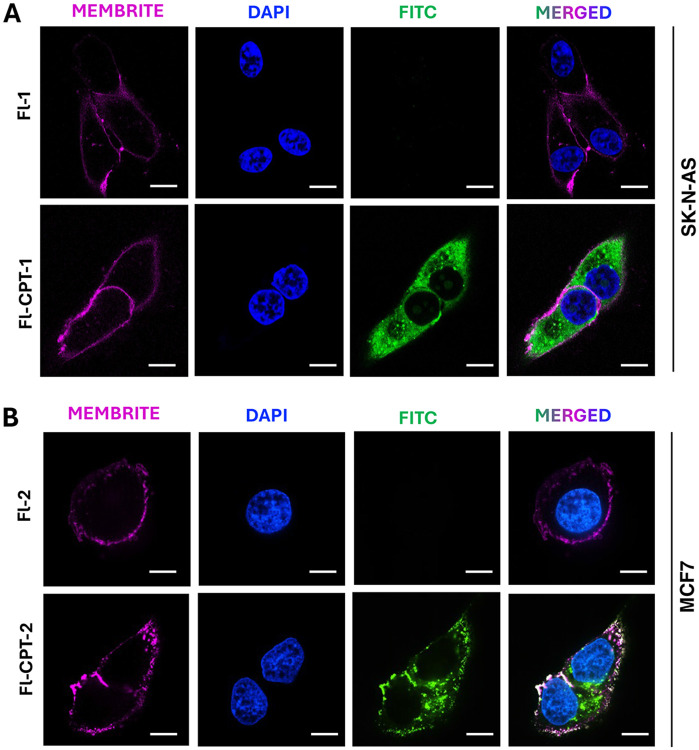
Subcellular localization of CPT conjugated peptides. **(A)** SK-N-AS cells were treated with 5 μM FL-**1** or FL-CPT-**1** for 16h. Cell membrane were stained using the Membrite Fix 640/660 cell surface staining kit, and nuclei were counterstained with DAPI. Scale bar, 10 μm. **(B)** MCF7 cells were treated with 5 μM FL-**2** or FL-CPT-**2** for 16 h. Cell membrane were stained using the Membrite Fix 640/660 cell surface staining kit, and nuclei were counterstained with DAPI. Scale bar, 10 μm.

We report a synthetic molecular scaffold called cell penetrating thyclotide (CPT) that effectively delivers peptides into cells (Fig. 1A). CPTs are constructed from a series of amide-linked monomers containing chiral tetrahydrofuran (THF) groups and non-natural polar sidechains (Fig.1B). The sidechains can be easily varied to optimize for cell uptake. Using solid phase peptide synthesis, the CPT monomer units are linked together and easily conjugated to bioactive peptides. Two previously reported peptide inhibitors (peptides **1** and **2**, along with their fluorescein-labeled derivatives Fl-**1** and Fl-**2**, Fig. 1C) were selected for detailed investigations into CPT-promoted cell uptake. In both cases, the peptide inhibitors had been optimized to bind their targets *in vitro* but lacked bioavailability in cells.^28,29^ CPT conjugates of the peptides yielded CPT-**1** and CPT-**2**, and the fluorescein-labeled versions of the conjugates (Fl-CPT-**1** and Fl-CPT-**2**) were also prepared. We initially attached a range of fluorescein-labeled CPTs with different sidechains to **1**, which is a polar peptide that modestly inhibits the enzyme SetD8 (a histone methyl-transferase that is overexpressed in several cancers).^30-32^ From this initial screen (Fig. S1A and B), we identified a piperidine sidechain in the CPT that optimally promotes cellular uptake. When SK-NA-S cells were treated with 5 μM Fl-CPT-**1** for 24 hours, imaging flow cytometry (Amnis ImageStreamX Mark II) (Fig. S1A and B) and confocal microscopy demonstrated remarkably efficient cellular uptake and diffuse distribution throughout the cytosol (Fig. 2A). Approximately 99% of SK-N-AS cells were positive for Fl-CPT-**1**, and among these, 77% exhibited predominantly cytoplasmic localization and 23% showed nuclear distribution (Fig. S2A). In addition, the median fluorescence intensity (MFI) was significantly higher than in cells treated with Fl-**1**. In the absence of CPT, FL-**1** also demonstrated relatively high cellular uptake (85.7% FITC positive cells); however, its MFI was significantly lower than that of Fl-CPT-**1** (Fig. S2, B and C), indicating that only small amounts of FL-**1** enter cells. To determine whether CPT-1 is localized to early endosomes following cellular uptake, SK-N-AS cells were transfected with CellLight^™^ Early Endosomes-RFP (Rab5a, BacMam 2.0) for 24 h and subsequently incubated with CPT-1 for an additional 1 h. Immunofluorescence analysis showed no detectable co-localization between CPT-1 and the early endosomal marker Rab5a, indicating that CPT-1 is not retained within early endosomes after internalization (Fig. S2D). Although FL-CPT-1 was efficiently internalized without endosomal entrapment, we were unable to observe consistent biological activity for CPT-**1** in cell-based assays possibly due to the modest inhibitory activity or rapid degradation of the peptide once inside the cell. The same CPT was next attached to peptide **2**, a hydrophobic peptide that has been highly optimized as an inhibitor of MDM2 binding to p53.^29^ Overexpression of MDM2 is associated with several cancers and has been a frequent target for therapeutic intervention.^33-35^ The small molecule Nutlin-3 also inhibits MDM2 and is a clinical candidate for the treatment of cancers, but undesired toxic side-effects in clinical trials have limited its development.^36-39^ *In vitro*, peptide **2** binds very strongly to MDM2 and its homolog MDMX, yet it has weak activity in cell-based assays.^29^ We confirmed that fluorescein-labeled peptide **2** (Fl-**2**) does not enter cells (Fig. S2E). In contrast, CPT-**2** conjugates dramatically overcome the inherently poor bioavailability of peptide **2**. As shown in Figure 2B, the fluorescein-labeled version of CPT-**2** (Fl-CPT-**2**) is able penetrate into MCF7 cells with diffuse distribution across the cytosol. Approximately 98% of MCF7 cells internalized the Fl-CPT-**2**, which was predominantly localized in the cytoplasm and partially in the nucleus. In contrast, the Fl-**2** lacking the CPT moiety exhibited minimal cellular uptake, with only approximately 25% of cells showing detectable fluorescence (Fig. S2E). Consistent with these observations, the MFI was significantly higher in cells treated with the Fl-CPT-**2** compared to those treated with the Fl-**2** (Fig. S2, F and G). Similar to CPT-1, CPT-2 also showed no detectable co-localization with the early endosomal marker Rab5a following cellular uptake (fig. S2H). These findings indicate that the CPT moiety may facilitate efficient escape from early endosomes independent of the peptide sequence.

## Bioavailability of CPT-2 in cancer cells

**Figure 3.**
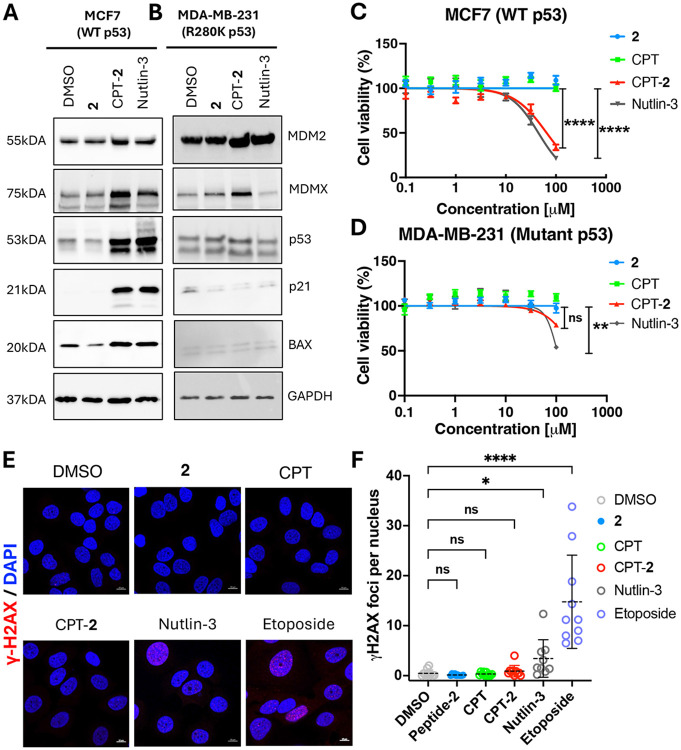
CPT-2 reduces cell viability through wild-type p53-dependent pathways *in vitro*. **(A, B)** Western blot analysis of protein expression in MCF7 **(A)** and MDA-MB-231 **(B)** cells following treatment with the indicated compounds at 10 μM for 24 h. Representative immunoblots showing modulation of p53-associated signaling pathways are presented. **(C, D)** Cell viability of MCF7 **(C)** and MDA-MB-231 **(D)** cells following treatment with the indicated compounds at concentrations ranging from 0.1 to 100 μM for 72 h, as determined by MTS assay. Data are presented as mean ± SEM from three independent biological replicates. Statistical significance was determined using the Kruskal-Wallis test followed by Dunn’s multiple comparisons test. P < 0.05 considered statistically significant. ****P ≤ 0.0001, **P ≤ 0.01, ns; not significant. **(E)** Immunofluorescence analysis of γ-H2AX foci formation in MCF7 cells following treatment with the indicated compounds at 50 μM for 96 h, except for Nutlin-3, which was used at 10 μM. Nuclei were counterstained with DAPI. Increased γ-H2AX staining indicates induction of DNA damage response pathways. Scale bar, 10 μm. **(F)** γ-H2AX foci per nucleus in MCF7 cells across treatment groups. Foci were quantified in Fiji (ImageJ). Each dot represents the mean number of γ-H2AX foci per nucleus for one image (10 images per group; 98 (DMSO), 88 (Peptide-**2**), 107 (CPT), 64 (CPT-2), 68 (Nutlin-3) and 70 (Etoposide) nuclei analyzed per group). Data were analyzed by Kruskal-Wallis test followed by Dunn’s multiple comparisons test versus the DMSO control. ****P ≤ 0.0001, *P= 0.0465, ns; not significant.

The biological activity of CPT-**2** was examined in detail to confirm that the molecule inhibits the MDM2-p53 interaction and leads to reactivation of p53. Treatment of MCF7 breast cancer cells, which have wild type p53, with 10 μM of CPT-**2** results in upregulation of p53 and induction of its downstream targets p21 (which leads to cell cycle arrest) and Bax (a proapoptotic protein). The molecule Nutlin-3 was used as a positive control and shows similar effects. In contrast, **2** alone has no effect. Modest increases in MDM2 and MDMX expression were also observed following CPT-**2** treatment (Fig. 3A), also consistent with the activity of Nutlin-3. To demonstrate specificity, control cells (MDA-MB-231) with a R280K mutation in p53 were used. The p53 mutation in the these cells prevents DNA binding and reactivation of p21 and BAX. Therefore, inhibition of MDM2 in MDA-MB-231 cells will not promote increases in p21 or Bax as p53 is unable to function due to the mutation. After confirming that Fl-CPT-**2** was similarly taken up by MDA-MB-231 cells (Fig. S3, A and C), treatment with CPT-**2** and Nutlin-3 both show no effects on the p21 and BAX protein levels (Fig. 3B). To further assess whether stabilized p53 accumulates in the nucleus together with MDM2, we performed a proximity ligation assay (PLA) in treated MCF7 cells. Fl-CPT-2 treatment for 24 h induced prominent nuclear PLA foci, indicating increased p53-MDM2 co-localization compared to the DMSO control (fig. S4, A and B). Because Fl-CPT-**2** does not localize to the nucleus, these foci reflect the accumulation of both interaction partners rather than a direct action of Fl-CPT-**2**. Inhibition of the MDM2/MDMX-p53 interaction stabilizes p53, which translocates to the nucleus and transcriptionally upregulates MDM2 through the p53-MDM2 autoregulatory feedback loop. The increase in proximity signal therefore reflects the combined nuclear accumulation of stabilized p53 and feedback-induced MDM2, which was also reported for Nutlin-3^40,41^.

Cytotoxicity assays at 72 h demonstrated that CPT-**2** selectively reduced the viability of MCF7 cells, with efficiency similar to Nutlin-3. In contrast, peptide-**2** and CPT alone (S10, Supporting Info, Table S3) showed no measurable effects (Fig. 3C). As expected, MDA-MB-231 cells were mostly unaffected by the same series of molecules except for high concentrations of Nutlin-3 that indicated non-specific toxicity (Fig. 3D). To evaluate potential off-target genotoxicity, we quantified nuclear γH2AX foci after 96 h treatment of MCF7 cells with **2**, CPT-**2**, or control molecules at 50 μM. With CPT-**2**, CPT, and **2**, foci counts were indistinguishable from the DMSO vehicle (Fig. 3, E and F). In contrast, the topoisomerase II inhibitor etoposide induced a pronounced increase in γH2AX foci (P < 0.0001), while Nutlin-3 showed a modest but significant increase (P < 0.05). Under the same western blotting conditions, CPT-**2** increased p53, p21 and Bax to levels comparable to Nutlin-3 and etoposide, yet did not elevate γH2AX (Fig. S5). Collectively, these results demonstrate that CPT-**2** activates p53 signaling through MDM2 inhibition in a wild-type p53-dependent manner while exhibiting reduced non-specific toxicity relative to Nutlin-3.

**Figure 4.**
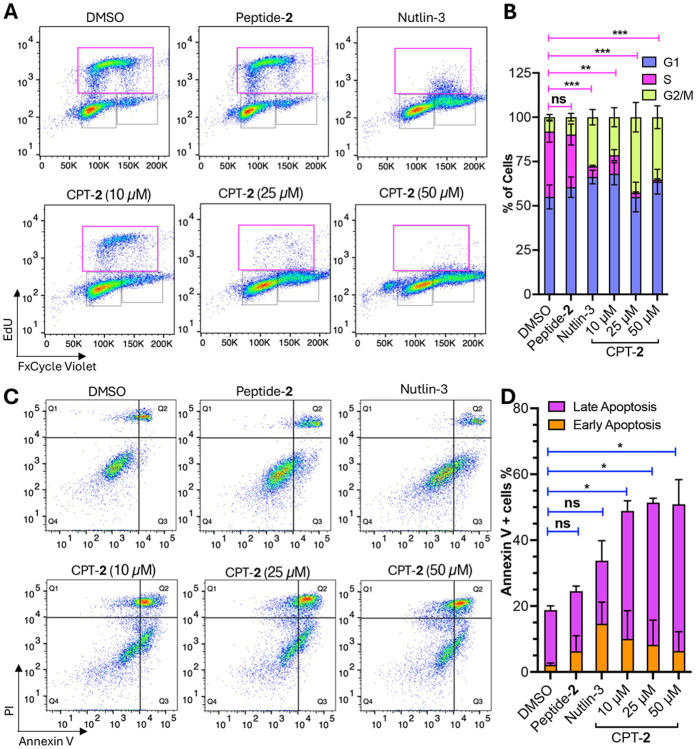
Effects of CPT-2 on cell cycle progression in MCF7 cells. **(A)** MCF7 cells were treated with DMSO (control), peptide-**2** (50 μM) alone, Nutlin-3 (10 μM), or increasing concentrations of CPT-**2** (10, 25, and 50 μM) for 24 h, followed by EdU incubation for an additional 2 h. Cell cycle distribution was analyzed by flow cytometry based on EdU incorporation (EdU-Alexa Fluor 488 staining) and DNA content (FxCycle Violet staining). Representative plots from three biological replicates are shown. **(B)** Quantification of the percentage of cells in each cell cycle phase following treatment. CPT-**2** induced an S-phase block in a concentration-dependent manner. Cell cycle distributions for the S phase were compared by two-way ANOVA followed by Dunnett’s multiple comparisons test versus vehicle (DMSO) control and are displayed above the bars; p ≤ 0.05 was considered significant. ***P≤ 0.001, **P= 0.0049, ns; not significant. **(C)** Representative flow cytometry plots of Annexin V-FITC and propidium iodide (PI) staining in MCF7 cells treated with DMSO, **2** (50 μM), Nutlin-3 (10 μM) or CPT-**2** (10, 25, or 50 μM) for 24 h. Cells were classified as viable (Q4, Annexin V^−^/PI^−^), early apoptotic (Q3, Annexin V^+^/PI^−^), late apoptotic (Q2, Annexin V^+^/PI^+^), or necrotic/dead (Q1, Annexin V^−^/PI^+^). **(D)** Percentage of early (Q3, Annexin V^+^/PI^−^) and late apoptotic cell (Q2, Annexin V^+^/PI^+^) populations in response to the indicated treatments. Late apoptotic cell (Q2, Annexin V^+^/PI^+^) populations were compared by two-way ANOVA followed by Dunnett’s multiple comparisons test versus vehicle (DMSO) control and are displayed above the bars; p ≤ 0.05 was considered significant. *P is 0.0452 (DMSO vs 10 μM), 0.0160 (DMSO vs 25 μM) and 0.0118 (DMSO vs 50 μM). ns; not significant.

The next series of experiments examined the effect of CPT-**2** on cell cycle distribution and apoptosis to distinguish whether the loss of proliferation caused by CPT-**2** reflects cell cycle arrest or cell death. Active DNA synthesis was assessed by 5-ethynyl-2’-deoxyuridine (EdU) incorporation combined with FxCycle Violet DNA content staining, allowing replicating (EdU^+^, S-phase) cells to be resolved from non-replicating G1 and G2/M populations. Cells treated with DMSO and **2** displayed a prominent EdU^+^ S phase population (36.9% and 29.7%, respectively), consistent with active proliferation. Treatment with CPT-**2** reduced the EdU^+^ S phase population, in a concentration dependent manner, to the following values: 10.1% at 10 μM, 2.4% at 25 μM, and 1.1% at 50 μM (Fig. 4A and B). The CPT-**2** induced depletion of S phase cells was accompanied by a significant accumulation of cells in G2/M (42.6% at 25 μM and 35.3% at 50 μM, relative to 8.1% in DMSO). The G1 population did not change significantly (Fig. 4B). Treatment with **2** alone was indistinguishable from DMSO across all phases. The cell cycle profile produced by CPT-**2** is similar to Nutlin-3, which reduced the S phase population to 6.1%. At the highest CPT-**2** dose (50 μM), there is a very low population of cells in the S phase. The depletion of S phase cells by CPT-**2**, but not by **2**, demonstrates that the anti-proliferative effect requires CPT conjugation. Furthermore, the effects induced by CPT-**2** are consistent with a p53 dependent mechanism of cell cycle arrest downstream of MDM2-p53 disruption.

To determine whether the cells were also undergoing apoptosis, MCF7 cells were co-stained with Annexin V and propidium iodide (PI) after 24h of treatment and analyzed by flow cytometry (Fig. 4C and D). CPT-**2** increased the Annexin V positive population relative to DMSO. At CPT-**2** concentrations of 10, 25, and 50 μM, early apoptotic cells (Q3, Annexin V^+^/PI^−^) were quantified at 10%, 8.2%, and 6.4%, while late apoptotic cells (Q2, Annexin V^+^/PI^+^) were 38.8%, 43.2%, and 44.5%, respectively (Fig. 4C and D). In contrast, Nutlin-3 did not produce a significant increase in early or late apoptotic cells. Surprisingly, the total Annexin V positive fraction remained largely constant (~49-51%) across the range of CPT-**2** concentrations; which contrasts with the results from cell-cycle arrest (Fig. 4B) and the loss of viability (Fig. 3C) which are dependent on CPT-**2** concentrations. It is possible that the Annexin V^+^/PI^+^ signal overestimates the true apoptotic fraction in this system. These findings indicate that engagement of the MDM2-p53 axis by CPT-**2** drives a predominantly cytostatic response that is concentration-dependent, and that cell death is more apparent at concentrations of 50 μM (Fig. 4C and D).

## Characterization of CPT cell uptake pathways

The chemical structure of CPT is very different from any other delivery vehicle that promotes cellular uptake. We therefore decided to investigate the pathways by which CPT-modified peptides enter cells. In the optimized CPT, the amines of the piperidine sidechains are likely protonated and positively charged under the aqueous conditions used for cell experiments. This series of positively charged secondary amines bears some resemblance to the frequent presence of lysine amino acids in cell penetrating peptides. The series of THF rings, however, are a very unexpected chemical feature that has not previously been used in delivery vehicles. To assess the contribution of THF rings to cellular uptake, a CPT analog lacking the THF groups (S11, Supporting Info, Table S3) was synthesized. This analog exhibited markedly reduced cellular uptake, with only ~17% of MCF7 cells showing uptake of S10, thereby demonstrating that the THF rings are critical for CPT activity (Fig. S6A). Interestingly, unconjugated Fl-CPT (S12, Supporting Info, Table S3) was also efficiently internalized by MCF7 cells, exhibiting uptake levels comparable to those of Fl-CPT-**2** (~95%). However, the MFI of Fl-CPT was significantly lower than that of Fl-CPT-**2** (Fig. S6B and C), indicating reduced intracellular accumulation despite similar uptake efficiency.

**Figure 5.**
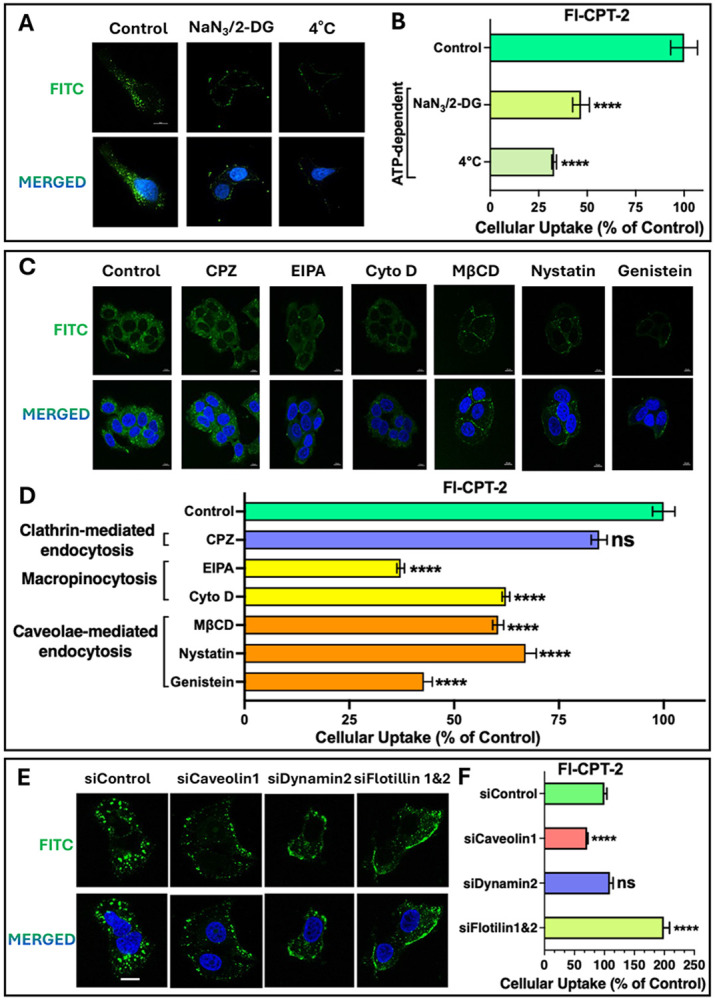
Mechanistic analysis of intracellular CPT-2 uptake in MCF7 cells. (**A**) Representative fluorescence microscopy images of MCF7 cells pretreated with NaN_3_/2-DG or incubated at 4°C, followed by treatment with 5 μM Fl-CPT-**2**. (**B**) Quantification of intracellular Fl-CPT-**2** uptake under the indicated conditions, normalized to DMSO-treated control cells and presented as percentage uptake relative to control. Statistical significance was determined using the Kruskal-Wallis test, with p < 0.05 considered statistically significant. ****P ≤ 0.0001. **(C)** Representative fluorescence microscopy images of MCF7 cells pretreated with endocytic inhibitors prior to incubation with 5 μM Fl-CPT-**2**. **(D)** Quantification of intracellular Fl-CPT-**2** uptake following treatment with the indicated endocytic inhibitors, normalized to DMSO-treated control cells. Statistical significance was determined using the Kruskal-Wallis test, with p < 0.05 considered statistically significant. ****P ≤ 0.0001, ns; not significant. **(E)** Representative fluorescence microscopy images of MCF7 cells transfected with the indicated siRNAs followed by treatment with 5 μM Fl-CPT-**2**. **(F)** Quantification of intracellular Fl-CPT-**2** uptake in cells transfected with the indicated siRNAs, normalized to control siRNA-treated cells. Statistical significance was determined using the Mann Whitney U test, with p < 0.05 considered statistically significant. ****P ≤ 0.0001, ***p=0.0002, ns; not significant.

Next, the cellular uptake of Fl-CPT-**2** was evaluated to determine whether internalization in MCF7 cells occurs through an active transport mechanism. MCF7 cells were pretreated with either a combination of sodium azide and 2-deoxyglucose or incubated at 4 °C for 30 min to inhibit energy-dependent uptake pathways, followed by incubation with Fl-CPT-**2** for 3 h. Both conditions reduced Fl-CPT-**2** uptake by more than 50%, indicating that cellular internalization requires an energy-dependent active transport mechanism (Fig. 5A and B).

Cells internalize extracellular molecules through several active endocytic pathways, including clathrin-mediated, caveolae-mediated, and flotillin-mediated endocytosis, as well as macropinocytosis.^42^ These pathways rely on distinct cellular components and regulatory factors for efficient uptake. Pharmacological inhibitors targeting specific pathway-associated proteins or processes can therefore be used to selectively disrupt individual internalization mechanisms. To determine which pathways are important for uptake of Fl-CPT-**2**, MCF7 cells were pretreated with an inhibitor for 30 min, followed by incubation with 5 μM of Fl-CPT-**2** for 1 h in the presence of an inhibitor. Immunofluorescence analysis demonstrated that inhibition of clathrin-mediated endocytosis with chlorpromazine (CPZ) slightly decreased (~15.4%) the cellular uptake of Fl-CPT-**2** (Fig. 5C and D), indicating a minimal contribution of this pathway to cellular internalization. In contrast, inhibition of macropinocytosis significantly decreased Fl-CPT-**2** uptake in MCF7 cells, with cytochalasin D and EIPA reducing uptake by >30% and ~60%, respectively. These findings suggest that macropinocytosis represents one of the major routes of Fl-CPT-**2** entry into cells, consistent with the enhanced uptake capacity of tumor cells for extracellular material.^43^ Inhibition of caveolae-mediated endocytosis markedly impaired Fl-CPT-**2** internalization as well. Treatment with methyl-β-cyclodextrin (MβCD), genistein, and nystatin reduced cellular uptake by approximately 39.5%, 33%, and 57%, respectively, supporting the involvement of lipid raft associated endocytic pathways in Fl-CPT-**2** uptake (Fig. 5C and D).

Because MβCD and nystatin disrupt cholesterol-rich lipid rafts that are involved in multiple endocytic pathways, additional validation experiments were performed using transient knockdown of caveolin-1, dynamin-2, and flotillin-1&2 in MCF7 cells for 72 h and followed by incubation with Fl-CPT-**2** (Fig. S7A). After knockdown of caveolin-1 (a key structural component required for caveolae formation), Fl-CPT-**2** uptake was reduced by approximately 29%, further supporting the contribution of caveolae-mediated internalization. In contrast, knockdown of dynamin-2 produced only a minimal decrease in uptake (~7.3%). Notably, silencing of flotillin-1 and flotillin-2 increased Fl-CPT-2 uptake by approximately 53%, suggesting that flotillin-dependent pathways are unlikely to assist with Fl-CPT-**2** internalization and may instead negatively regulate its uptake (Fig. 5E and F).^44^ These observations were all consistent with imaging flow cytometry analysis (fig. S7B and C).

## Discussion

CPTs are a unique and versatile chemical scaffold to promote the delivery of peptide cargo into cells. A benefit of the CPT platform is the ability to easily modify sidechains with different chemical functionalities that alter cellular uptake properties (Figure S1). After exploring the uptake of CPTs with 8 different sidechains, an optimized CPT was developed for the delivery of either a polar or a hydrophobic peptide. It is surprising that the chemical combination of THF and piperidine sidechains leads to a very efficient CPT for cellular uptake as neither chemical group has previously been associated with cell penetrating peptides. These molecular features represent a significant departure from long-established observations of the chemical properties that lead to cellular uptake of cell penetrating peptides and other oligomeric synthetic scaffolds. Guanidine and amine groups (usually presented from arginine or lysine amino acids) are usually considered critical chemical entities for cell uptake, and many cell penetrating peptides as well as other related types of synthetic scaffolds rely on the presence of multiple guanidine and amine groups to promote cell uptake.^45,46^ Yet, endosomal entrapment frequently limits the bioactivity of cargo molecules conjugated to polycationic delivery agents.^6^ After CPT conjugation to peptides **1** and **2**, significantly enhanced cell uptake without endosomal entrapment was clearly demonstrated. CPT-**1** entered cells, showed diffuse distribution throughout the cytoplasm, but did not demonstrate reliable bioactivity, possible due to instability of the peptide or weak inhibitory activity. Extensive studies with CPT-**2** show that cell uptake and biological activity is greatly enhanced compared to peptide **2** alone, demonstrating that CPT-**2** can both enter cells and sufficiently escape from endosomes to exert biological activity. Investigating the uptake pathway of CPT-**2** showed that energy-dependent endocytosis is necessary, and that a combination of both macropinocytosis and caveolar-mediated endocytosis are dominant pathways. Interestingly, clathrin-mediated endocytosis is not important for uptake.

Peptide **2** had been previously optimized to inhibit MDM2 and MDMX,^29^ two proteins often overexpressed in cancer cells which inhibit the activity of p53. Many studies have focused on disrupting MDM2 binding to p53, and several clinical candidates have been examined.^47^ Despite the very potent inhibitory activity of **2** in vitro, the lack of cellular uptake and bioavailability likely hindered the further development of this peptide. This situation is common with many bioactive peptides as well as larger molecules that are unable to enter cells on their own. By coupling an optimized CPT with **2**, cellular delivery and biological activity is greatly enhanced. Relative to a small molecule positive control (Nutlin-3), CPT-**2** is also less toxic. These beneficial properties could reignite further clinical development of **2** as its CPT conjugate. The strategy of using CPT to enhance cellular uptake and biological activity can be applied to other molecules, making this an exciting new approach to advance the therapeutic development of peptides and other classes of molecules that have low bioavailability.

## Supplementary Material

Supplement 1
