# Supplementary material for: Cell Penetrating Thyclotides Facilitate Efficient Delivery of Bioactive Peptides into Cells
Source: bioRxiv. 2026 Jul 2:2026.07.01.735572. Preprint. [Version 1] doi: 10.64898/2026.07.01.735572 (PMC13345009; doi:10.64898/2026.07.01.735572)
Supplement: Supplement 1 [file NIHPP2026.07.01.735572v1-supplement-1.pdf]

Supplementary Materials

Chemicals and antibodies

Chlorpromazine (CPZ; Sigma-Aldrich, Cat. C8138) was dissolve in sterile water at a stock concentration of 30 mg/ml. Cytochalasin D (Sigma-Aldrich, Cat. C8273) was dissolved in DMSO to prepare a 5 mM stock solution and used at a final concentration of 2 µM. EIPA (5-(N-ethyl-N-isopropyl) amiloride; Selleckchem, Cat. S9849, 10 mM in DMSO) used at a final concentration of 100 µM. Methyl-ß-cyclodextrin (MßCD; Sigma-Aldrich, Cat. C4555) was dissolved in sterile cell culture grade water and applied at a final concentration of 10mM. Genistein (Sigma-Aldrich, Cat. G6649) was dissolved in DMSO and used at a final concentration of 200 µM. Nutlin-3 (Selleckchem, Cat. S1061) was dissolved in DMSO and used at a final concentration of 10 µM as a positive control for MDM2 inhibition.

For siRNA transfection, we used caveolin-1 siRNA(h) (Santa Cruz, Cat. sc-29241), Dynamin II siRNA(h) (Santa Cruz, Cat. sc-35236), Flotillin-1 siRNA(h) (Santa Cruz, Cat. sc-35391), Flotillin-2 siRNA(h) (Santa Cruz, Cat. sc-35393) and Control siRNA (Invitrogen, #AM4611).

For western blot, we used p53 (Calbiochem, Cat. OP43L; 1:1000), MDM2 (Santa Cruz, Cat. sc-965; 1:200), MDMX (Proteintech #84534-6-RR), p21 (Santa Cruz, Cat. sc-187; 1:200), BAX (Cell Signaling, Cat. 5023S; 1:1000), GAPDH (Cell Signaling Technology, Cat. D4C6R; 1:1000), caveolin-1 (Cell Signaling Technology, Cat. 3267S; 1:1000), Dynamin II (Santa Cruz, Cat. sc-166669; 1:200), Flotillin-1 (Santa Cruz, Cat. sc-74566; 1:200), Flotillin-2 (Santa Cruz, Cat. sc-28320; 1:200), γ-H2AX (Cell Signaling Technology, Cat. 5438S; 1:1000). For PLA experiment, we used p53 (Calbiochem, Cat. OP43L; 1:100) and MDM2 (Cell Signaling Technology, Cat. 86934; 1:100).

Cell lines and cell culture

MCF7, MDA-MB-231 and SK-N-AS cells were purchased from American Type Culture Collection (ATCC, Manassas, VA, USA). All cell lines were maintained at 37 °C in a humidified incubator with 5% CO_2. MCF7 cells were cultured in EMEM supplemented with 10% FBS and 1% penicillin/streptomycin. SK-N-AS cells were cultured in DMEM supplemented with 10% FBS, 0.1 mM non-essential amino acids (NEAA), and 1% penicillin/streptomycin, while MDA-MB-231 cells were cultured in DMEM supplemented with 10% FBS and 1% penicillin/streptomycin. For treatments, all molecules were diluted in the respective complete growth medium for each cell line.

Imaging Flow Cytometry

Cells were seeded in 6-well plates and, after 24 h, treated with fluorescein-labeled CPT-peptides diluted in culture medium. 16 hours after treatment, the peptide-containing medium was removed, and cells were washed three times with ice-cold PBS to prevent further uptake. Cells were detached using trypsin-EDTA and centrifuged at 1500 rpm for 5 min. Pellets were resuspended in 50 µl PBS containing 2% FBS and 20 µM DRAQ5, a DNA-staining dye (Abcam, Cat. ab108410). Cell internalization was measured on a 2-camera/12-channel Amnis ImageStream Mark II imaging flow cytometer (Cytek Bioscience, Fremont, CA) at 40X magnification equipped with 4 lasers. Data were recorded in Channel 1 (bright field), channel 2 (FITC) and channel 11 (DRAQ5) for 10,000 single cells per sample. For each cell, bright field (Channel 1), FITC (Channel 2) and DRAQ5 (Channel 11) images were used for the analysis. IDEAS 6.3 software (Amnis Corp, Seattle, WA) was used to analyze cellular uptake and intracellular distribution.

Confocal Microscopy

MCF7 cells were seeded in µ-Slide 8-well chambers (Ibidi, Cat. 80806) at a density of 2 x10^4 cells per well in complete medium. After 24 h, cells were treated with 5 µM fluorescein-labeled CPT-peptides diluted in complete medium. Following incubation 1, 3 or 16 h, depending on the experiment, cells were washed three times with ice-cold PBS and stained using Biotium Membrite Fix 640/660 Cell Surface Staining Kit according to the manufacturer's instructions prior to fixation. Cells were then fixed with 4% formaldehyde (Thermo Fisher Scientific, Cat. 28906) for 15 min at room temperature, washed again with PBS, and mounted using SlowFade™ Diamond Antifade Mountant with DAPI (Thermo Fisher Scientific, Cat. S36973). For early endosome marker colocalization, cells were treated with CellLight BacMam 2.0 early endosome-RFP marker, Rab5a (Invitrogen, Carlbad, CA, USA) diluted in the culture medium. The endosome marker transduction was performed at a PPC of 50. Super-resolution images were acquired using either a Nikon SoRa spinning disk confocal microscope (Nikon Instruments Inc., Melville, NY, USA) equipped with a 60x apochromat oil immersion objective (NA 1.49) and a Hamamatsu ORCA Fusion BT sCMOS camera (Teledyne Photometrics, Tucson, AZ, USA), or a Zeiss LSM 880 confocal microscope with a 63x plan-apochromat oil immersion objective lens (NA 1.4) and Airyscan detector (Carl Zeiss Microscopy GmbH, Jena, Germany). Image deconvolution was performed using Nikon Elements software (v5.3) with a modified Richardson-Lucy iterative algorithm, or Zeiss ZEN Blue software for Airyscan processing.

Western blotting

MCF7 and MDA-MB-231 cells were seeded in 6-well plates and treated with 10 µM Peptide-**2**, CPT-**2** or Nutlin-3 for 24 h. Cells were then washed with PBS, trypsinized, and lysed in RIPA buffer (Thermo Fisher Scientific, Cat. 89901) supplemented with a protease inhibitor cocktail (Roche, Cat.11836153001). Lysates were sonicated for 5 min (30 sec on/30 sec off cycles) at 4°C, and protein concentrations were determined using the Bradford assay (Thermo Fisher Scientific, Cat. 23200). Equal amounts of protein (30 µg) were resolved on 4-20% Mini-PROTEAN TGX precast gels (Bio-Rad, Cat. 4561096) and transferred onto nitrocellulose membranes (Bio-Rad, Cat. 1704159). Membranes were blocked for 1 hour in 5% non-fat milk prepared in TBS with 0.1% Tween-20 (TBS-T), then incubated overnight at 4°C with primary antibodies diluted in the blocking buffer. After three washes with TBS-T, membranes were incubated with HRP-conjugated secondary antibodies for 1 hour at room temperature. Protein bands were visualized using SuperSignal™ West Femto Maximum Sensitivity Substrate (Thermo Fisher Scientific, Cat. 34096) with a ChemiDoc MP Imaging System (Bio-Rad) and analyzed using Image Lab software (v6.1).

Cytotoxicity Assay

Cell viability was determined using the Promega CellTiter 96® AQueous One Solution Cell Proliferation Assay (MTS) following the manufacturer's protocol. Cells were seeded in 96-well tissue culture plates and treated the next day with DMSO, peptide-**2**, CPT alone, or CPT-**2** at serial concentrations ranging from 0.1 to 100 µM. After 72 hours of incubation, MTS reagent was added directly to each well and the plates were incubated for an additional 3 hours at 37 °C with 5% CO_2. Absorbance was measured at 490 nm using an Omega 640 spectrophotometer. Cell viability was calculated relative to DMSO-treated controls and averaged from three replicates.

Proximity ligation assay (PLA)

PLA was performed using Duolink® In Situ Detection Reagents Red (Sigma, Cat. DUO92008-100RXN) according to the manufacturer's instructions. Briefly, 2.5 $x10^4$ MCF7 cells were seeded on Millicell EZ slides (Sigma, Cat. PEZGS0816) and cultured for 24 h. Cells were fixed with 4% paraformaldehyde in PBS for 15 min, permeabilized with 0.3% Triton X-100 for 10 min, and blocked with Duolink blocking solution at 37 °C for 1 h. Samples were then incubated overnight at 4 °C with primary antibodies against p53 and MDM2. Ligation and amplification were performed according to the manufacturer's protocol. The ligation step was performed for 30 min at 37 °C, followed by polymerization reaction for 100 min at 37 °C. The cells were mounted with mounting media with DAPI. Fluorescent signals were imaged using a Zeiss LSM 880 confocal microscope.

Cell uptake assessment with Endocytosis Inhibitors

MCF7 cells were seeded in μ-Slide 8-well chambers (Ibidi, #80806) at a density of 2 $x10^4$ cells per well in complete growth medium and cultured for 24 hours. The following day, the medium was replaced with fresh complete medium containing 0.1% DMSO or specific endocytosis inhibitors, and the cells were preincubated for 30 minutes. Subsequently, fluorescence-labeled CPT-**2** was added to the cultures in the presence or absence of inhibitors. After 1 hour of incubation, the cells were washed three times with ice-cold PBS and processed for either confocal microscopy or flow cytometry analysis. For quantitative analysis, cells were segmented as nuclear and cytoplasm and quantified using a custom script written in Phyton (v.3.10), utilizing the Cellpose deep-learning AI package (PMID: 39939718). Custom segmentation models were trained using the NIH HPC Biowulf cluster (http://hpc.nih.gov)

Cell Uptake assessment with siRNA Transfection

All siRNAs were purchased from Santa Cruz Biotechnology and are listed in section "Chemicals and Antibodies". MCF7 cells were seeded in 6-well plates at a density of 1 $x10^5$ cells per well in complete growth medium and cultured for 24 hours. siRNA transfection was performed using Lipofectamine RNAiMAX (Invitrogen, Cat. #13778075) according to the manufacturer's instructions, at a final siRNA concentration of 10 nM. After 72 hours of transfection, the cells were incubated with fluorescence-labeled CPT-**2** at 37 °C for 1 hour, washed three times with ice-cold PBS, and processed for confocal microscopy or flow cytometry analysis. Quantitative analysis was performed using the same experimental conditions as those applied for the cell uptake assay with endocytosis inhibitors.

Statistical analysis

Statistical analyses were performed using GraphPad Prisim 11. Data are presented as mean ± SEM. Comparisons between two or more groups were performed with Mann-Whitney, Kruskal Wallis or two-way ANOVA followed by Dunnett's multiple comparisons test. A p-value < 0.05 was considered statistically significant. The *, **, ***, and **** symbols indicate $P \leq 0.05$, $P \leq 0.01$, $P \leq 0.001$, and $P \leq 0.0001$, respectively.

Supplementary Tables and Figures

Table S1. Mass characterization data for peptides and peptide-thyclotide conjugates (SETD8)[a]

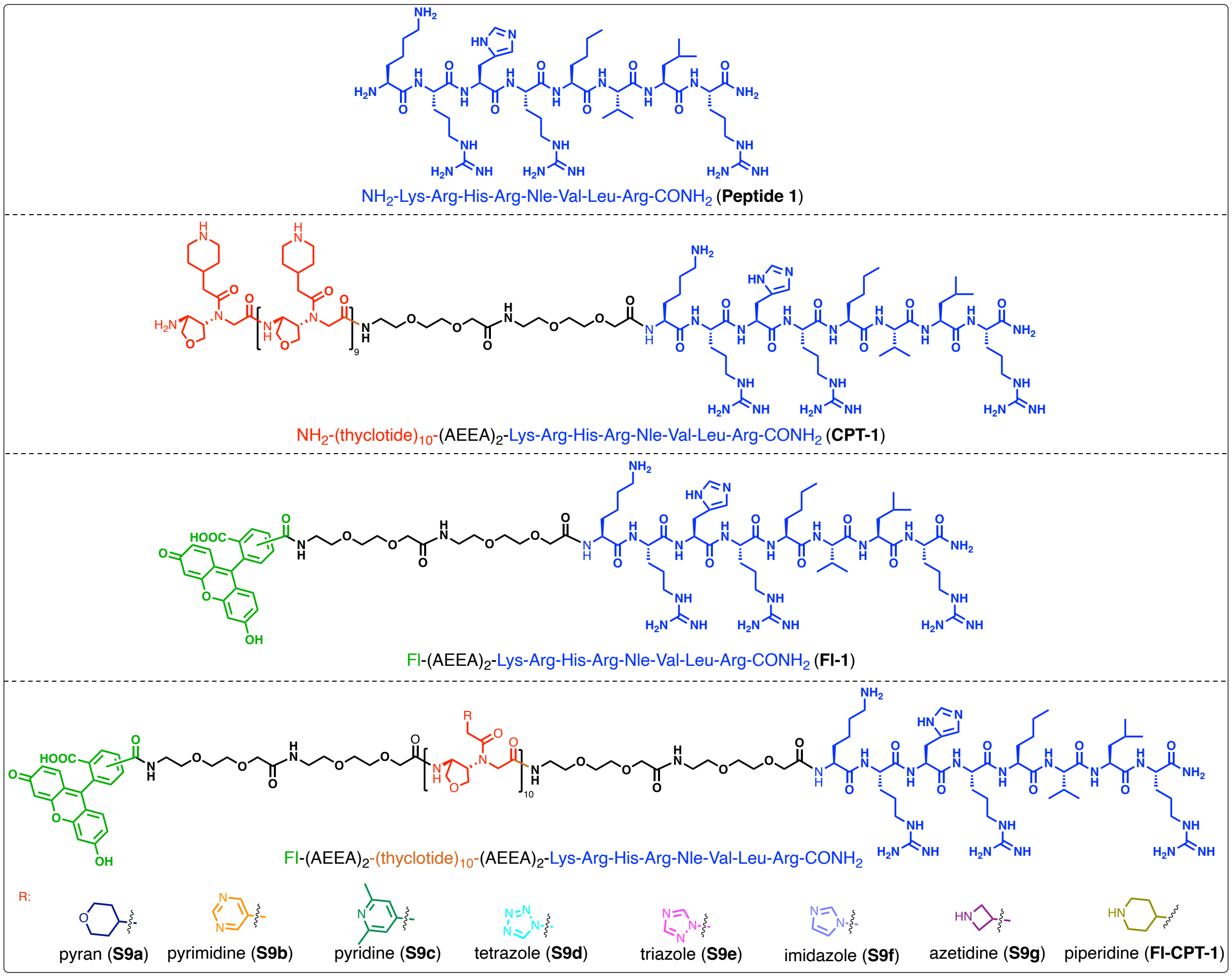

entry	Peptide-thyclotide conjugate/Peptide	calculated	observed
1	Peptide (**Peptide 1**)	359.6	359.6
2	Peptide-thyclotide conjugate (**CPT-1**)	831.7	831.7
3	Fl-labelled peptide (**Fl-1**)	575.7	575.7
4	Fl-labelled peptide-pyran thyclotide conjugate (**S9a**)	1566.8	1566.8
5	Fl-labelled peptide-pyrimidine thyclotide conjugate (**S9b**)	1546.7	1546.7
6	Fl-labelled peptide-pyridine thyclotide conjugate (**S9c**)	1636.8	1636.8
7	Fl-labelled peptide-tetrazole thyclotide conjugate (**S9d**)	1513.0	1513.4
8	Fl-labelled peptide-triazole thyclotide conjugate (**S9e**)	1510.0	1510.0
9	Fl-labelled peptide-imidazole thyclotide conjugate (**S9f**)	1506.7	1506.7
10	Fl-labelled peptide-azetidine thyclotide conjugate (**S9g**)	1470.1	1470.3
11	Fl-labelled peptide-piperidine thyclotide conjugate (**Fl-CPT-1**)	1563.6	1563.6

[a]Fl = 5/6-fluorescein, AEEA = 2-(2-aminoethoxy)ethoxyacetyl group. All data in this table correspond to the corresponding quintuply charged ion $[M+TFA+5H]^{5+}$ (for **CPT-1**), or triply charged ion $[M+3H]^{3+}$ (for other peptides and peptide-thyclotide conjugates).

Table S2. Mass characterization data for peptides and peptide-thyclotide conjugates (MDM2)[a]

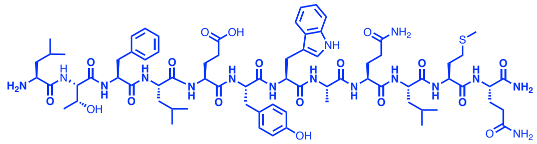

NH_2-Leu-Thr-Phe-Leu-Glu-Tyr-Trp-Ala-Gln-Leu-Met-Gln-$CONH_2$ (**Peptide 2**)

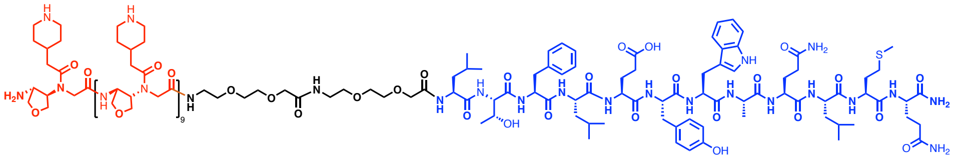

NH_2-(thyclotide)$_{10}$-(AEEA)$_2$-Leu-Thr-Phe-Leu-Glu-Tyr-Trp-Ala-Gln-Leu-Met-Gln-$CONH_2$ (**CPT-2**)

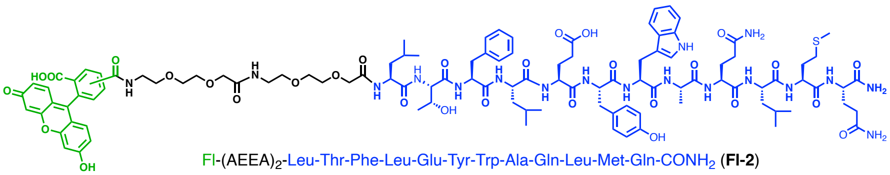

Fl-(AEEA)$_2$-Leu-Thr-Phe-Leu-Glu-Tyr-Trp-Ala-Gln-Leu-Met-Gln-$CONH_2$ (**Fl-2**)

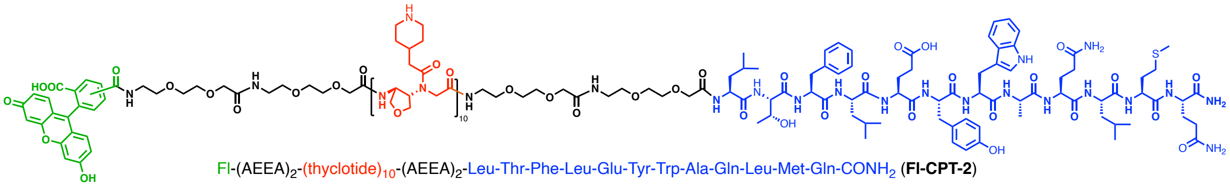

Fl-(AEEA)$_2$-(thyclotide)$_{10}$-(AEEA)$_2$-Leu-Thr-Phe-Leu-Glu-Tyr-Trp-Ala-Gln-Leu-Met-Gln-$CONH_2$ (**Fl-CPT-2**)

entry	Peptide-thyclotide conjugate/Peptide	calculated	observed
1	Peptide (**Peptide 2**)	771.4	771.4
2	Peptide-thyclotide conjugate (**CPT-2**)	901.9	901.9
3	Fl-labelled peptide (**Fl-2**)	1096.0	1096.0
4	Fl-labelled peptide-thyclotide conjugate (**Fl-CPT-2**)	1031.6	1031.6

[a]Fl = 5/6-fluorescein, AEEA = 2-(2-aminoethoxy)ethoxyacetyl group. All data in this table correspond to the corresponding quintuply charged ion $[M+5H]^{5+}$ (for **CPT-2** and **Fl-CPT-2**), or doubly charged ion $[M+2H]^{2+}$ (for **Peptide 2** and **Fl-2**).

Table S3. Mass characterization data for negative control molecules[a]

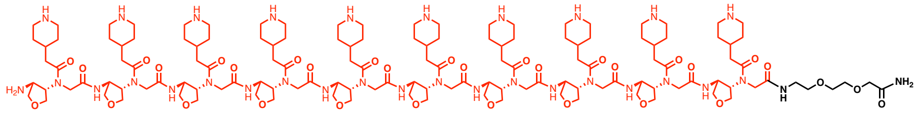

NH_2-(thyclotide)$_{10}$-AEEA-$CONH_2$ (**S10**)

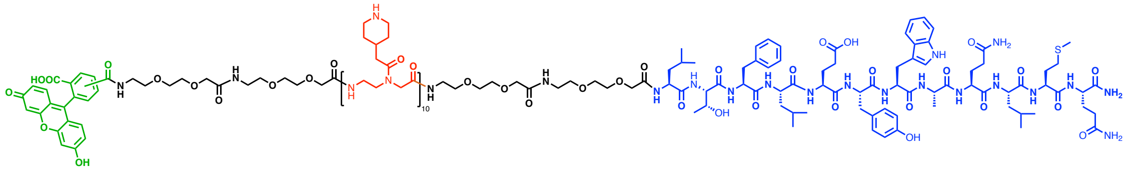

Fl-(AEEA)$_2$-(ethylenediamine-glycine-piperidine)$_{10}$-(AEEA)$_2$-Leu-Thr-Phe-Leu-Glu-Tyr-Trp-Ala-Gln-Leu-Met-Gln-$CONH_2$ (**S11**)

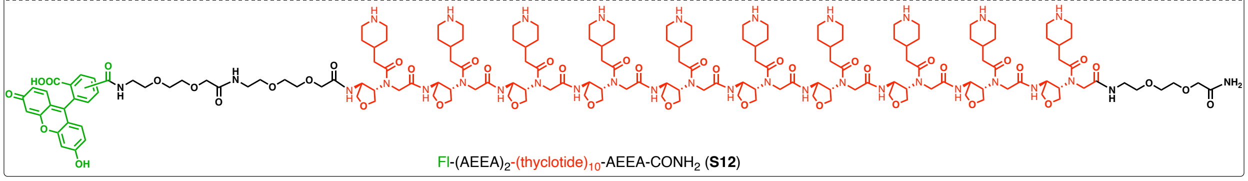

Fl-(AEEA)$_2$-(thyclotide)$_{10}$-AEEA-$CONH_2$ (**S12**)

entry	Peptide-thyclotide conjugate/Thyclotide	calculated	observed
1	Cell penetrating thyclotide (CPT) (**S10**)	709.7	709.8
2	Fl-labelled peptide acyclic piperidine conjugate (**S11**)	947.5	947.5
3	Fl-labelled cell penetrating thyclotide (**S12**)	697.6	697.6

[a]Fl = 5/6-fluorescein, AEEA = 2-(2-aminoethoxy)ethoxyacetyl group. All data in this table correspond to the corresponding quintuply charged ion $[M+5H]^{5+}$ (for **S11** and **S12**), or quadruply charged ion $[M+4H]^{4+}$ (for **S10**).

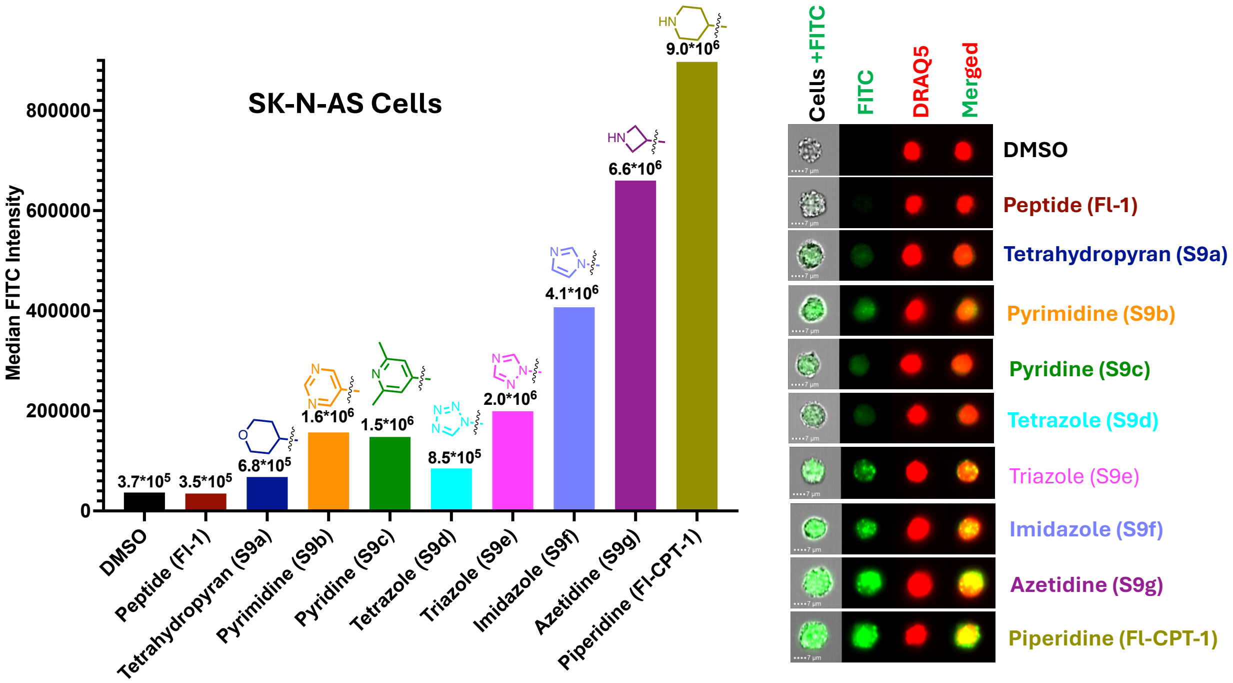

Fig. S1. Screening of CPT conjugates containing different functional moieties. (A) SK-N-AS cells were treated with 5 µM peptide-**1** conjugated to different CPT derived moieties and analyzed by imaging flow cytometry to evaluate intracellular uptake based on median fluorescence intensity (MFI). **(B)** Representative imaging flow cytometry images of cells treated with each conjugate. Nuclei were stained with DRAQ5. Scale bar, 7 µm.

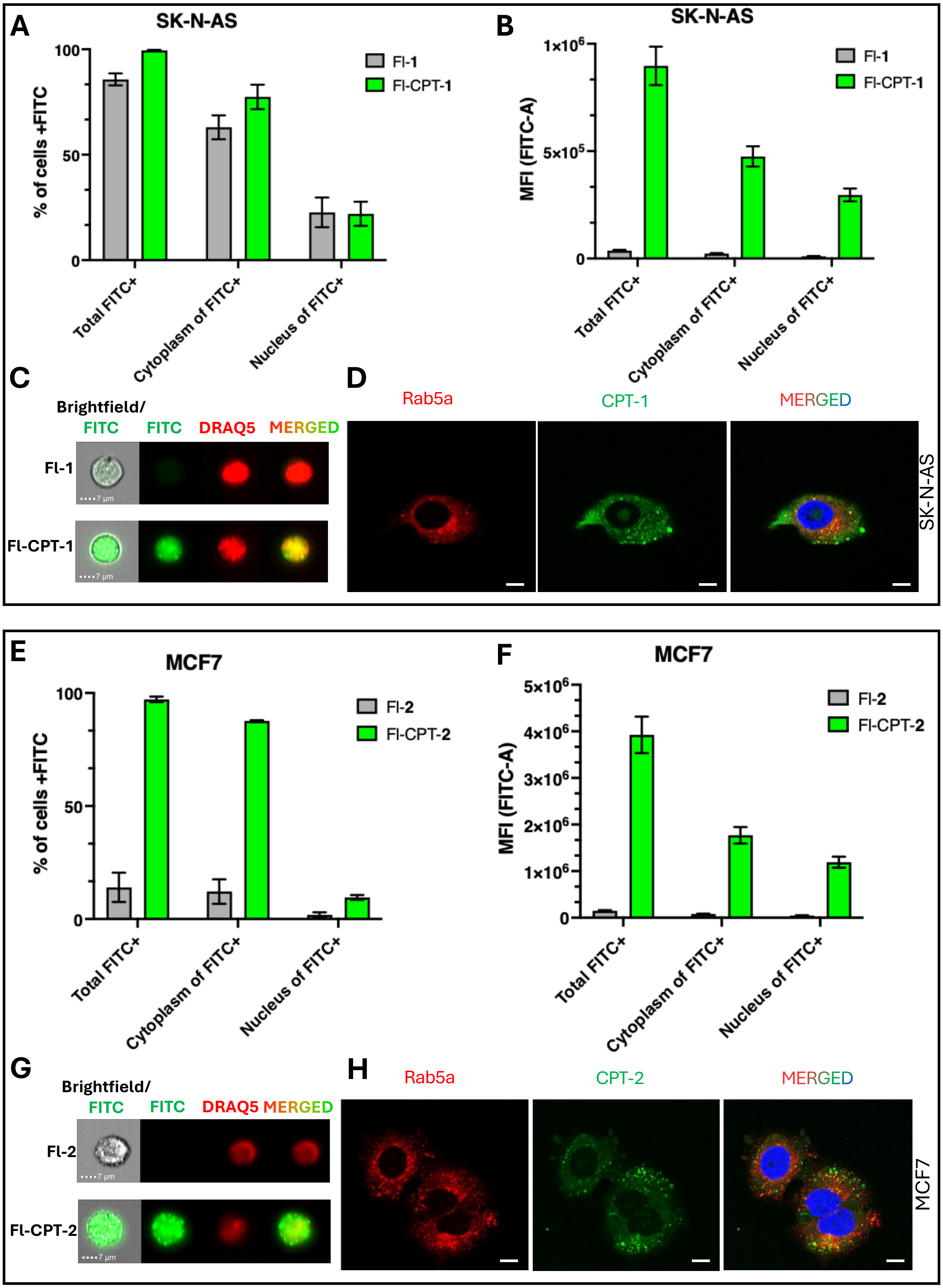
A
SK-N-AS
% of cells +FITC
100
50
0
Fl-1
Fl-CPT-1
Total FITC+
Cytoplasm of FITC+
Nucleus of FITC+
B
SK-N-AS
MFI (FITC-A)
1×10^6
5×10^5
0
Fl-1
Fl-CPT-1
Total FITC+
Cytoplasm of FITC+
Nucleus of FITC+
C
Brightfield/ FITC
FITC
DRAQ5
MERGED
Fl-1
Fl-CPT-1
D
Rab5a
CPT-1
MERGED
SK-N-AS
E
MCF7
% of cells +FITC
100
50
0
Fl-2
Fl-CPT-2
Total FITC+
Cytoplasm of FITC+
Nucleus of FITC+
F
MCF7
MFI (FITC-A)
5×10^6
4×10^6
3×10^6
2×10^6
1×10^6
0
Fl-2
Fl-CPT-2
Total FITC+
Cytoplasm of FITC+
Nucleus of FITC+
G
Brightfield/ FITC
FITC
DRAQ5
MERGED
Fl-2
Fl-CPT-2
H
Rab5a
CPT-2
MERGED
MCF7

Fig. S2. Cellular uptake efficiency of CPT-peptide conjugates. (A) Percentage of SK-N-AS cells positive for internalized Fl-**1** or Fl-CPT-**1** following treatment with 5 µM compounds for 16 h, as determined by imaging flow cytometry. **(B)** Quantification of intracellular uptake based on median fluorescence intensity (MFI). **(C)** Representative imaging flow cytometry images of SK-N-AS cells treated with Fl-**1** or Fl-CPT-**1**. Nuclei were stained with DRAQ5. **(D)** SK-N-AS cells were transfected with Rab5a (CellLight Early Endosomes-RFP) 24 h prior to incubation with Fl-CPT-**1** for 1 h. Nuclei were counterstained with DAPI. Scale bar, 10 µm. **(E)** Percentage of MCF7 cells positive for internalized Fl-**2** or Fl-CPT-**2** following treatment with 5 µM compounds for 16 h, as determined by imaging flow cytometry. **(F)** Quantification of intracellular uptake based on median fluorescence intensity (MFI). **(G)** Representative imaging flow cytometry images of cells treated with Fl-**2** or Fl-CPT-**2**. Nuclei were stained with DRAQ5. **(H)** MCF7 cells were transfected with Rab5a (CellLight Early Endosomes-RFP) 24 h prior to incubation with Fl-CPT-**2** for 1 h. Nuclei were counterstained with DAPI. Scale bar, 10 µm.

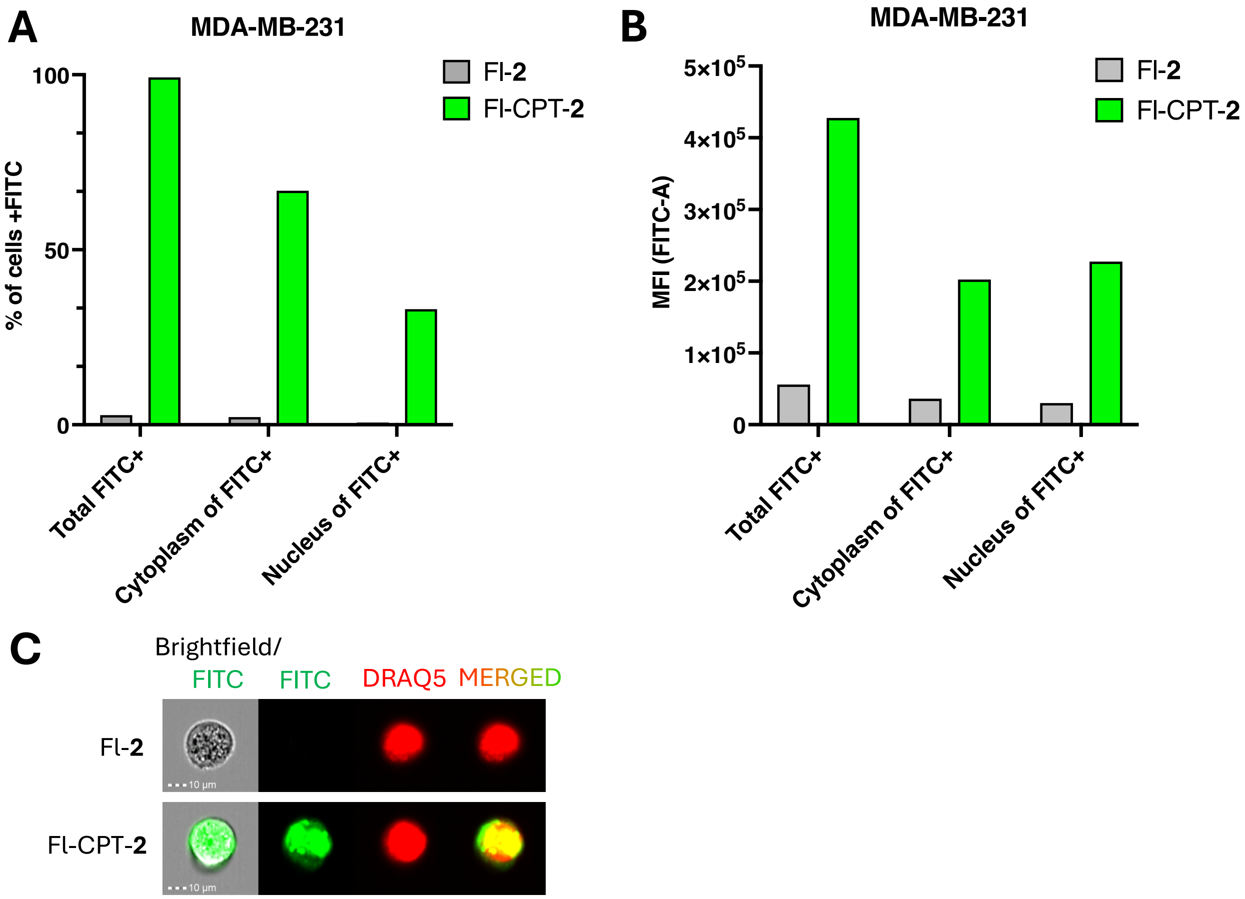

Fig. S3. Cellular uptake efficiency of CPT-peptide-2 on MDA-MB-231 cells. (A) Percentage of MDA-MB-231 cells positive for internalized Fl-**2** or Fl-CPT-**2** following treatment with 5 µM compounds for 16 h, as determined by Amnis imaging flow cytometry. **(B)** Quantification of intracellular uptake based on median fluorescence intensity (MFI). **(C)** Representative imaging flow cytometry images of cells treated with Fl-**2** or Fl-CPT-**2**. Nuclei were stained with DRAQ5.

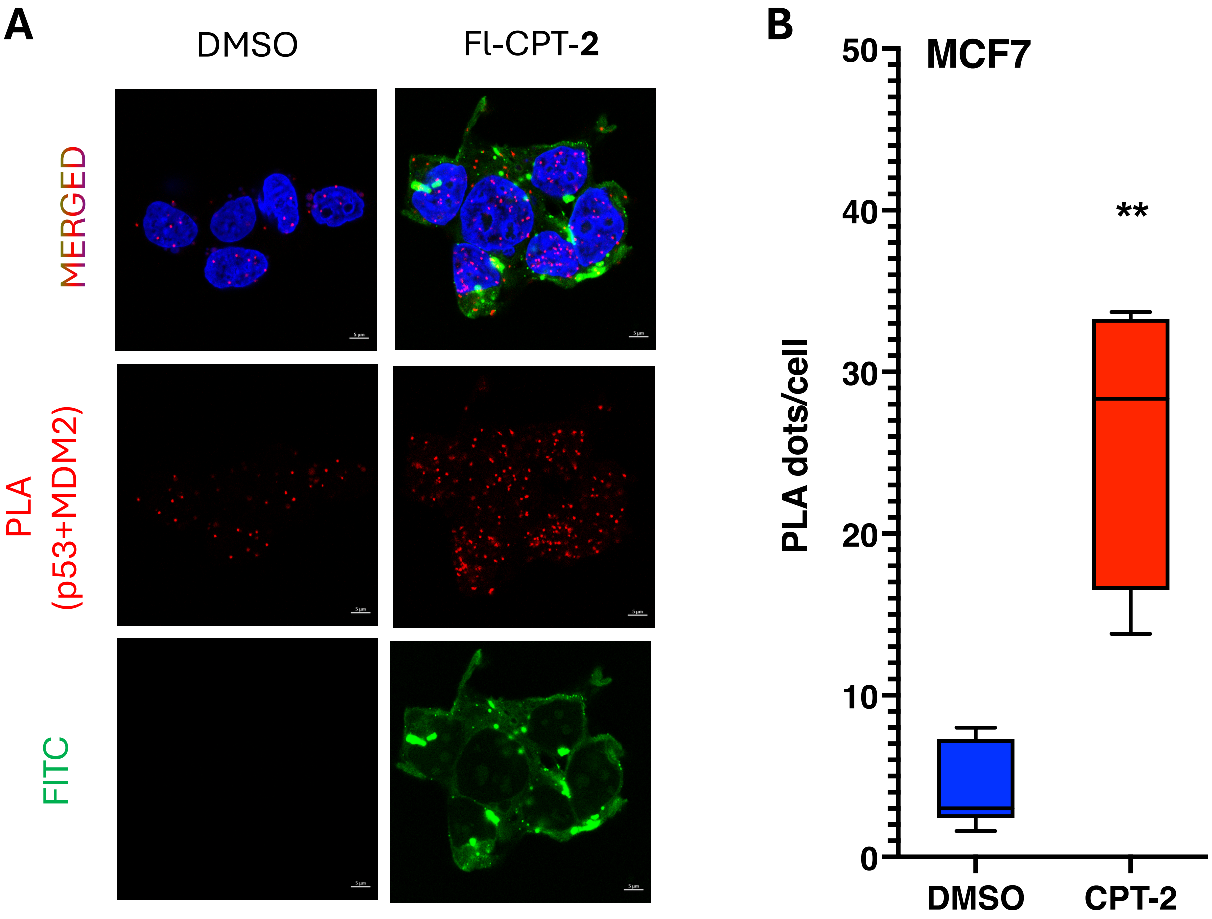

Fig. S4. CPT-2 enhances nuclear p53-MDM2 interaction in MCF7 cells. (A) Proximity ligation assay (PLA) of MCF7 cells treated with DMSO or 10 µM CPT-**2** for 24 h. Red fluorescent puncta indicate close proximity and interaction between p53 and MDM2 within the nucleus. **(B)** Quantification of p53-MDM2 interactions in cells treated with DMSO or CPT-**2**. Data are presented as mean ± SD from 10 analyzed images from each sample. Statistical significance was determined using the Mann-Whitney test; $**p < 0.01$.

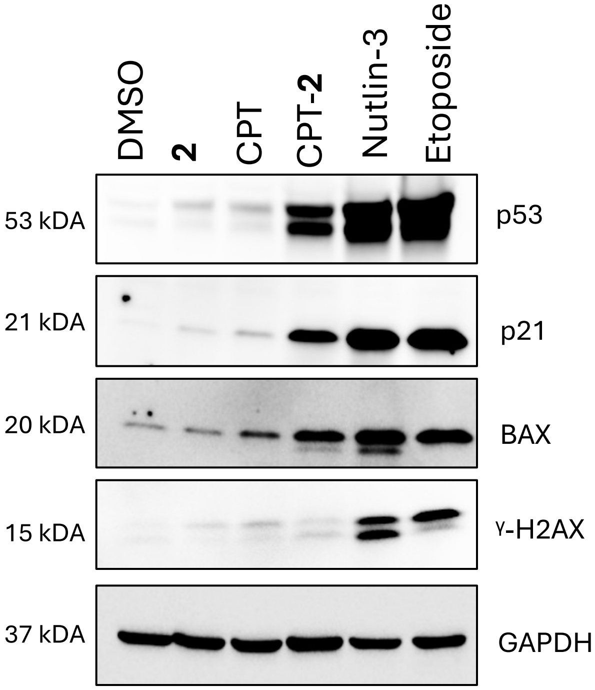

Fig. S5. CPT-2 does not induce DNA damage response. Western blot analysis of MCF7 cells treated with the indicated compounds at 50 μM for 96 h, except for Nutlin-3, which was used at 10 μM. Representative western blots demonstrate changes in protein expression associated with prolonged treatment. Increased γ-H2AX staining indicates induction of DNA damage response pathways.

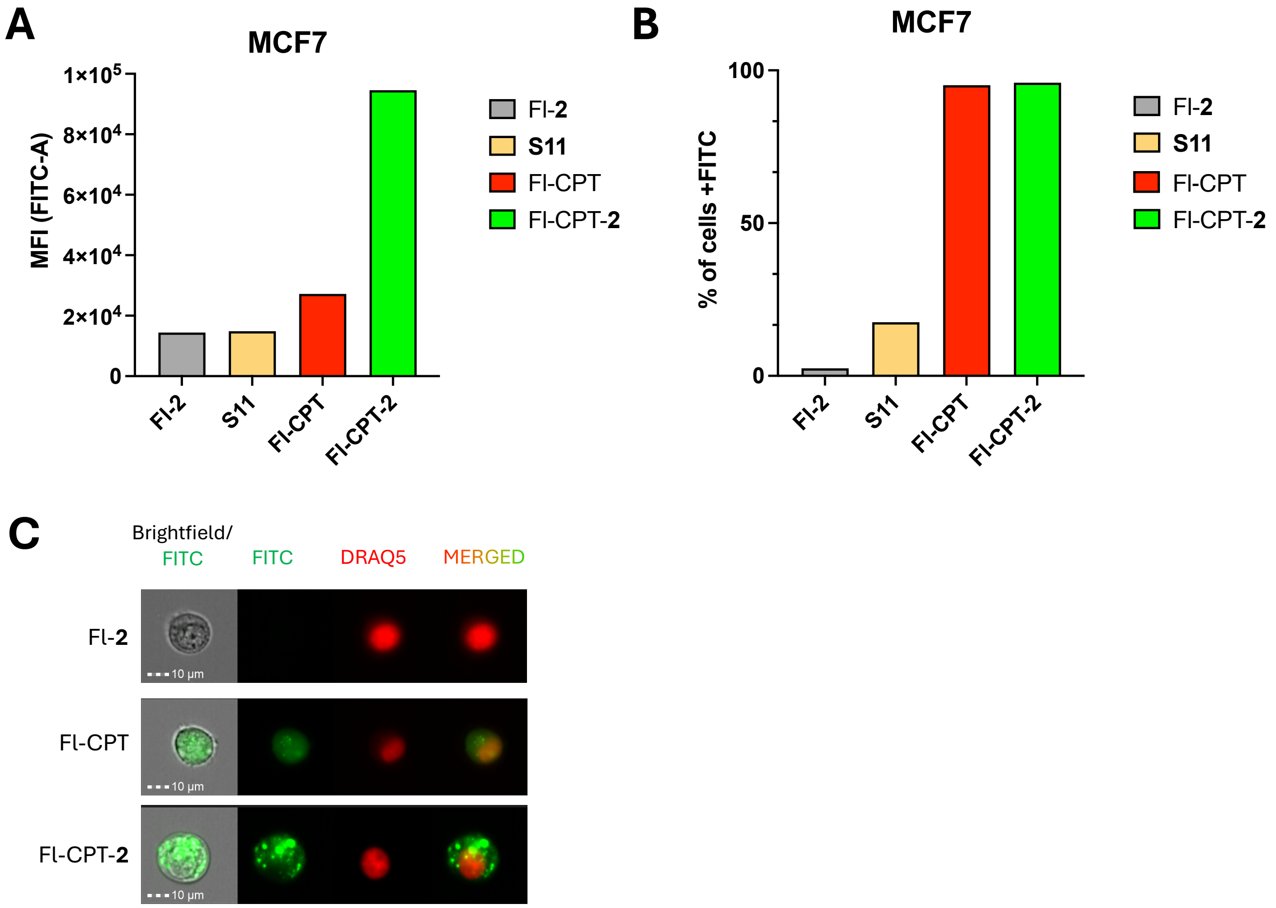

Fig. S6. THF ring is critical for cellular uptake. (A) Percentage of MCF7 cells positive for internalized for Fl-**2**, **S11**, Fl-CPT and FL-CPT-**2** following treatment with 5 µM compounds for 16 h, as determined by Amnis imaging flow cytometry. **(B)** Quantification of intracellular uptake based on median fluorescence intensity (MFI). **(C)** Representative imaging flow cytometry images of cells treated with Fl-**2,** Fl-CPT (**S12**) or Fl-CPT-**2**. Nuclei were stained with DRAQ5.

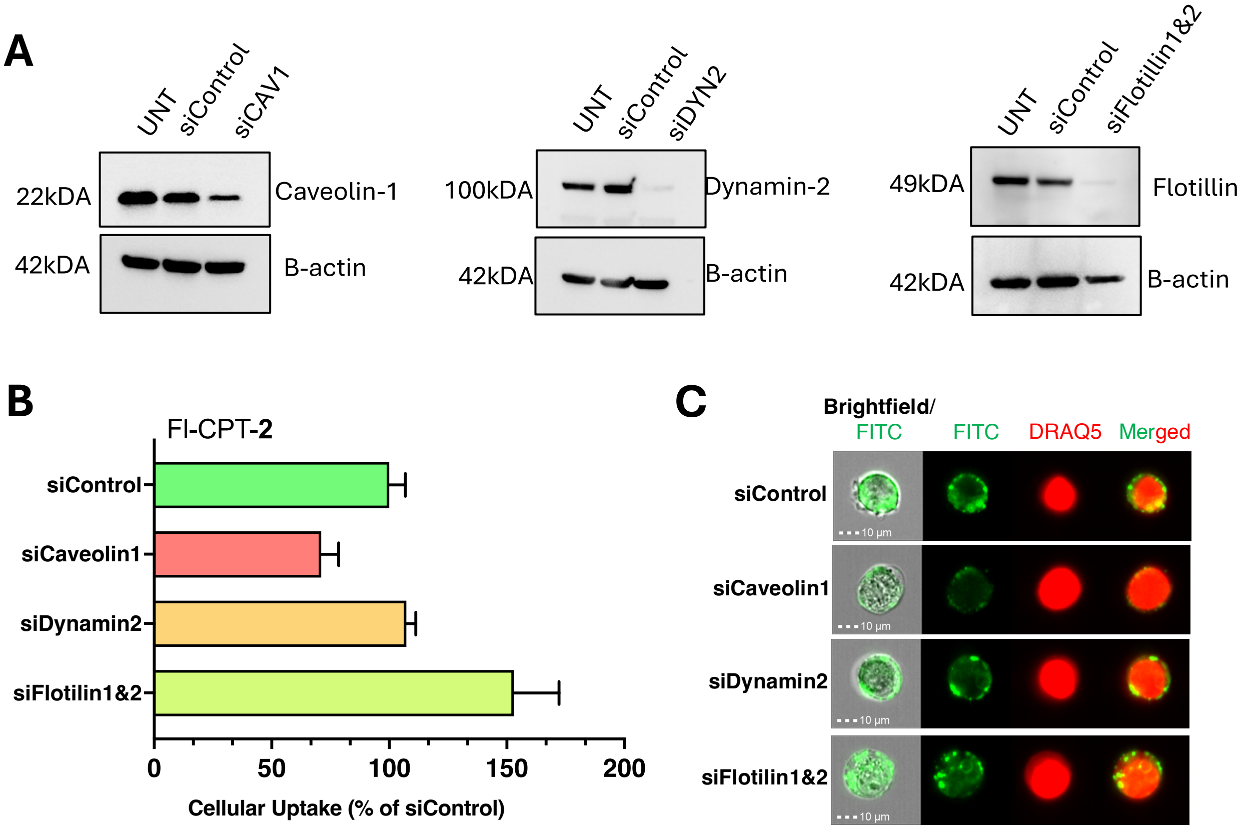

Fig. S7. Assessment of lipid raft-mediated endocytosis following siRNA-mediated knockdown of target genes. (A) MCF7 cells were transiently transfected with 10 nM gene-specific siRNAs for 72 h. Knockdown efficiency was validated by western blot analysis using antibodies against Caveolin-1, Dynamin-2, Flotillin-1&2, and β-actin as a loading control. **(B)** Quantification of intracellular Fl-CPT-**2** uptake under the indicated knockdown conditions. Cellular uptake was analyzed by Amnis imaging flow cytometry, normalized to siControl-transfected cells, and expressed as the percentage relative to siControl. **(C)** Representative imaging flow cytometry images showing intracellular fluorescence intensity of Fl-CPT-**2** in MCF7 cells transfected with siControl, siCaveolin-1, siDynamin-2, or siFlotillin-1&2 for 72 h, followed by incubation with Fl-CPT-**2** for 1 h.
